# Carboxylation Capacity Can Limit C_3_ Photosynthesis at Elevated CO_2_ throughout Diurnal Cycles

**DOI:** 10.3390/plants10122603

**Published:** 2021-11-27

**Authors:** James Bunce

**Affiliations:** Adaptive Cropping Systems Laboratory, USDA-ARS, Beltsville, MD 20705-2350, USA; buncejames49@gmail.com

**Keywords:** photosynthesis, elevated CO_2_, Rubisco, electron transport, light, diurnal cycle

## Abstract

The response of carbon fixation in C_3_ plants to elevated CO_2_ is relatively larger when photosynthesis is limited by carboxylation capacity (V_C_) than when limited by electron transport (J). Recent experiments under controlled, steady-state conditions have shown that photosynthesis at elevated CO_2_ may be limited by V_C_ even at limiting PPFD. These experiments were designed to test whether this also occurs in dynamic field environments. Leaf gas exchange was recorded every 5 min using two identical instruments both attached to the same leaf. The CO_2_ concentration in one instrument was controlled at 400 μmol mol^−1^ and one at 600 μmol mol^−1^. Leaves were exposed to ambient sunlight outdoors, and cuvette air temperatures tracked ambient outside air temperature. The water content of air in the leaf cuvettes was kept close to that of the ambient air. These measurements were conducted on multiple, mostly clear days for each of three species, *Glycine max*, *Lablab purpureus*, and *Hemerocallis fulva*. The results indicated that in all species, photosynthesis was limited by V_C_ rather than J at both ambient and elevated CO_2_ both at high midday PPFDs and also at limiting PPFDs in the early morning and late afternoon. During brief reductions in PPFD due to midday clouds, photosynthesis became limited by J. The net result of the apparent deactivation of Rubisco at low PPFD was that the relative stimulation of diurnal carbon fixation at elevated CO_2_ was larger than would be predicted when assuming limitation of photosynthesis by J at low PPFD.

## 1. Introduction

Photosynthesis by terrestrial vegetation is a large component of the annual global carbon balance, and predicting how the photosynthetic CO_2_ assimilation (A) of terrestrial vegetation will respond to increased concentrations of CO_2_ in the atmosphere is vital to understanding the future global carbon cycle [1,2,3]. Plants with C_3_ photosynthetic metabolism are of predominant importance in terms of numbers of species, total CO_2_ fixation, use as food for humans, and the responsiveness of photosynthesis to projected changes in atmospheric CO_2_. Predictions of photosynthesis of C_3_ plants at elevated CO_2_ concentrations often use the Farquhar–von Caemmerer–Berry biochemical model of photosynthesis [4] and its recent modifications [5].

In the Farquhar–von Caemmerer–Berry model of C_3_ photosynthesis [4], A at high photosynthetic photon flux density (PPFD) is limited by the maximum carboxylation capacity of Rubisco (V_Cmax_) at low CO_2_ concentrations, by maximum electron transport capacity (J_max_) at higher CO_2_, and sometimes by the rate of utilization of triose phosphates (TPU) at the highest CO_2_ concentrations [5], with all of these rate-limiting parameters having different temperature dependencies. Because TPU limitation did not occur in these experiments, the focus here will be on V_Cmax_ and J_max_ at saturating PPFD, and V_C_ and J at limiting PPFD. The external CO_2_ at the transition between limitation by V_Cmax_ and J_max_ at high PPFD varies among species, and with implementations of the FvCB model, from about 450 to 700 μmol mol^−1^ [3], so the issue is highly relevant to global change issues. Experiments exposing plants to elevated CO_2_ have sometimes found photosynthesis at elevated CO_2_ at high PPFD to be limited by V_Cmax_ and sometimes by J_max_, depending upon species and level of elevated CO_2_ (reviewed in [6]).

**Figure 1:**
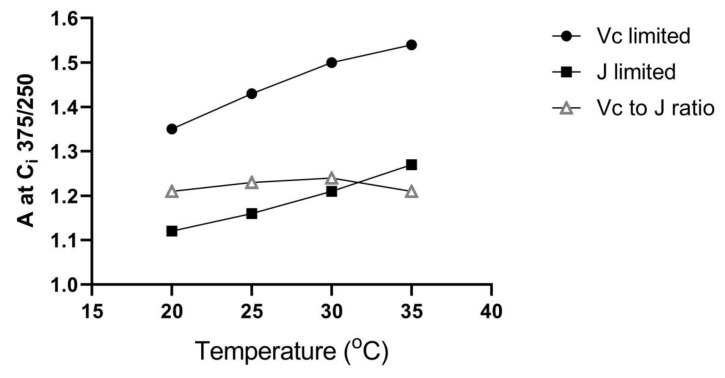
Hypothetical light-saturated rates of CO_2_ assimilation (A) at internal CO_2_ concentration (C_i_) = 375 μmol mol^−1^ relative to those measured at C_i_ = 250 μmol mol^−1^ as a function of temperature, for leaves where A is limited by carboxylation capactity (V_c_) or electron transport (J), and the ratio of these two values of A, using the Farquhar–von Caemmerer–Berry model [4].

The relative increase in A caused by increased CO_2_ concentration inside the leaf (C_i_) is less at a given temperature when A is limited by J than when limited by V_C_ [3,4]. This is illustrated in Figure 1, which shows that for an increase in C_i_ from 250 to 375 μmol mol^−1^ (i.e., approximate C_i_ for ambient and 1.5 × ambient CO_2_), A is stimulated about 20% more when A is limited by V_C_ than when limited by J, over a wide range of temperatures.

**Figure 2:**
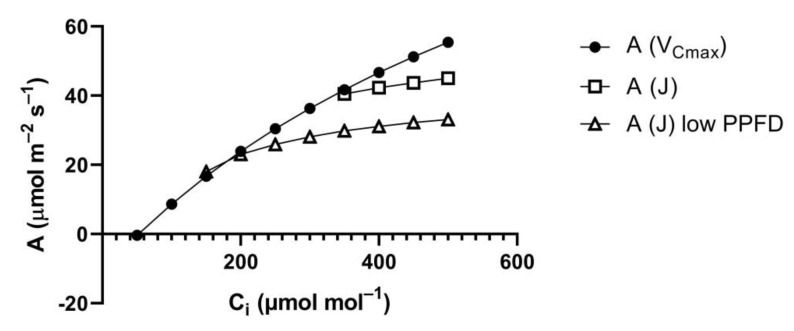
Hypothetical examples of assimilation rates (A) vs. internal CO_2_ concentrations (C_i_) at saturating and low PPFD, as predicted by the FvCB photosynthesis model [4]. Rates of A based on limitation by V_Cmax_ and by J at high and low PPFD are given. In all cases, actual A is the minimum of the A (V_Cmax_) and A (J) curves.

At lower PPFD, it is often assumed that carboxylation capacity remains constant while electron transport rate decreases. This primarily lowers A at higher C_i_ (Figure 2) and would result in a smaller relative stimulation of A between a C_i_ of 250 and 375 μmol mol^−1^, for example [7]. However, the assumption of constant carboxylation capacity at lower PPFD is incorrect, in the steady-state [8]. Bunce [8] found that the initial slope of A vs. C_i_ curves, an in situ measure of Rubisco carboxylation capacity, decreased as PPFD decreased in the steady-state, in a range of C_3_ species measured, over a range of temperatures. The result was that the relative stimulation of A at elevated vs. ambient CO_2_ did not decrease as PPFD decreased, at any temperature [8].

The purpose of these experiments was to determine whether the same apparent decrease in V_C_ (deactivation of Rubisco) at low PPFD observed in the steady-state also occurred under naturally varying PPFD in dynamic field environments in C_3_ species. This was tested by determining whether A at elevated CO_2_ during different parts of diurnal cycles was better predicted from A at ambient CO_2_ using the assumption of limitation of A at elevated CO_2_ by Vc or by J.

## 2. Results

**Figure 3:**
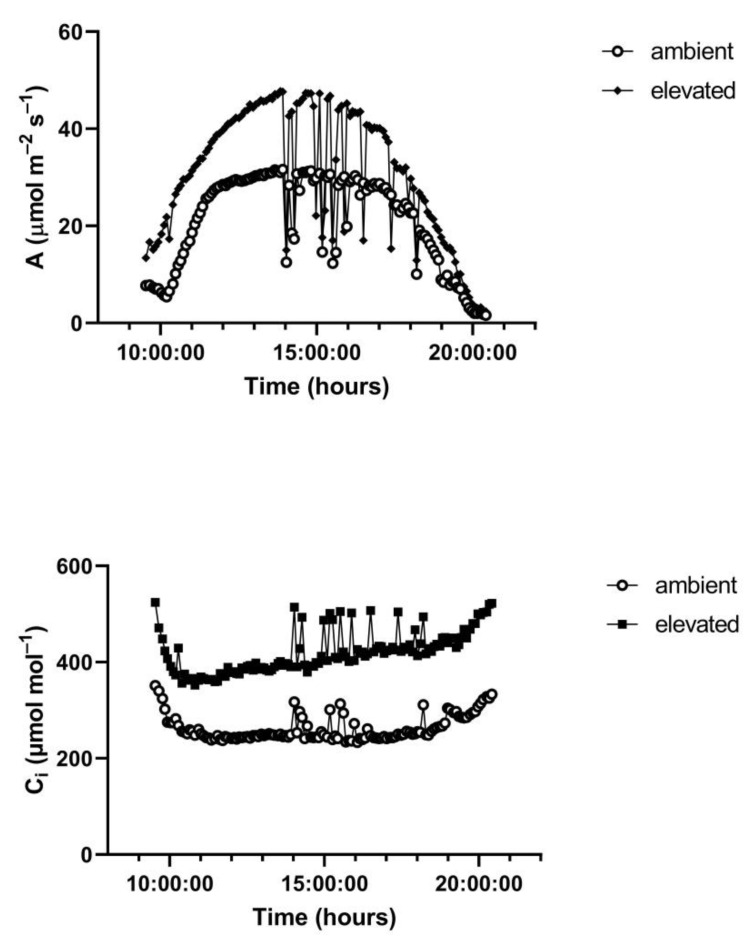

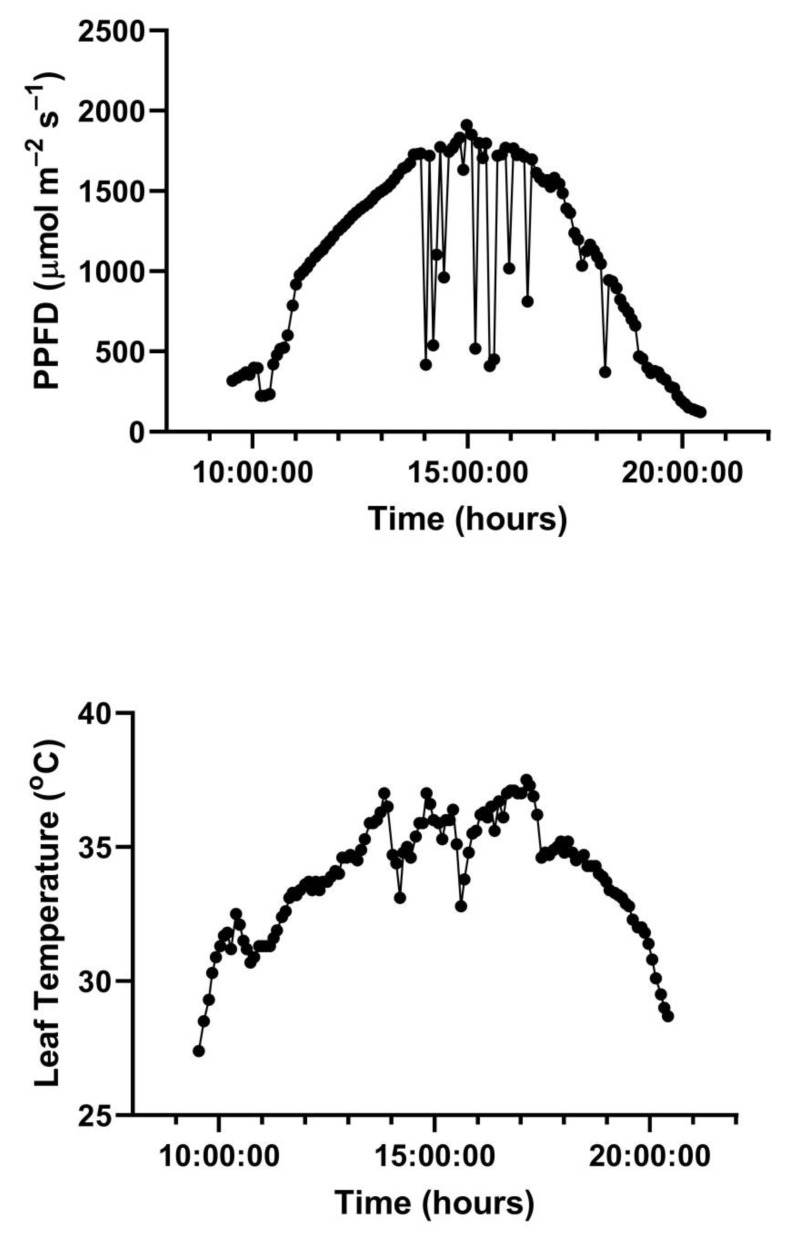
Diurnal patterns of PPFD, leaf temperature, A, and C_i_ of paired leaves of *G. max* kept at 400 (ambient) and 600 (elevated) μmol mol^−1^ CO_2_. Environmental variables are given for only one of the two leaflets, for clarity. See text for details.

**Figure 4:**
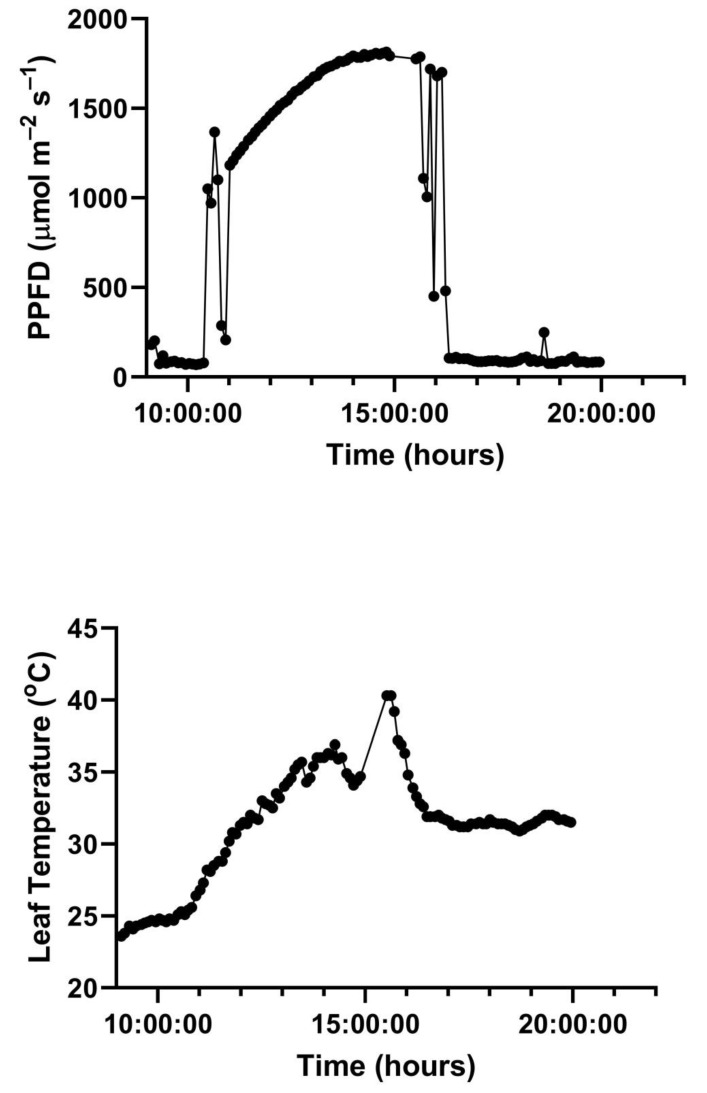

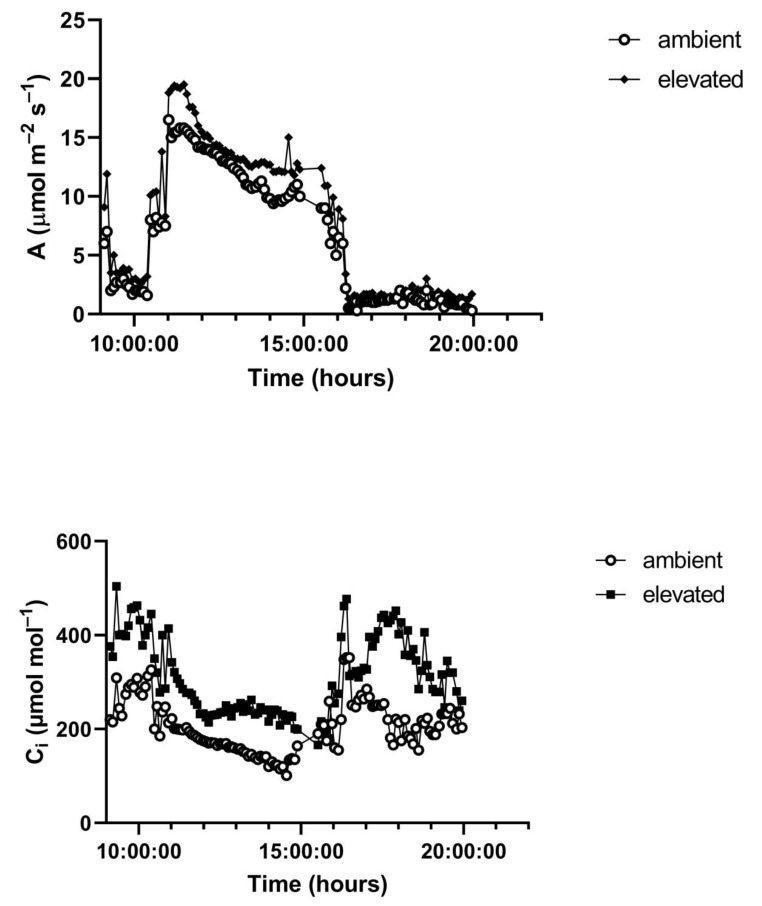
Diurnal patterns of PPFD, leaf temperature, A, and C_i_ of paired leaves of *L. purpureus* kept at 400 (ambient) and 600 (elevated) μmol mol^−1^ CO_2_. Environmental variables are given for only one of the two leaflets, for clarity. See text for details.

**Figure 5:**
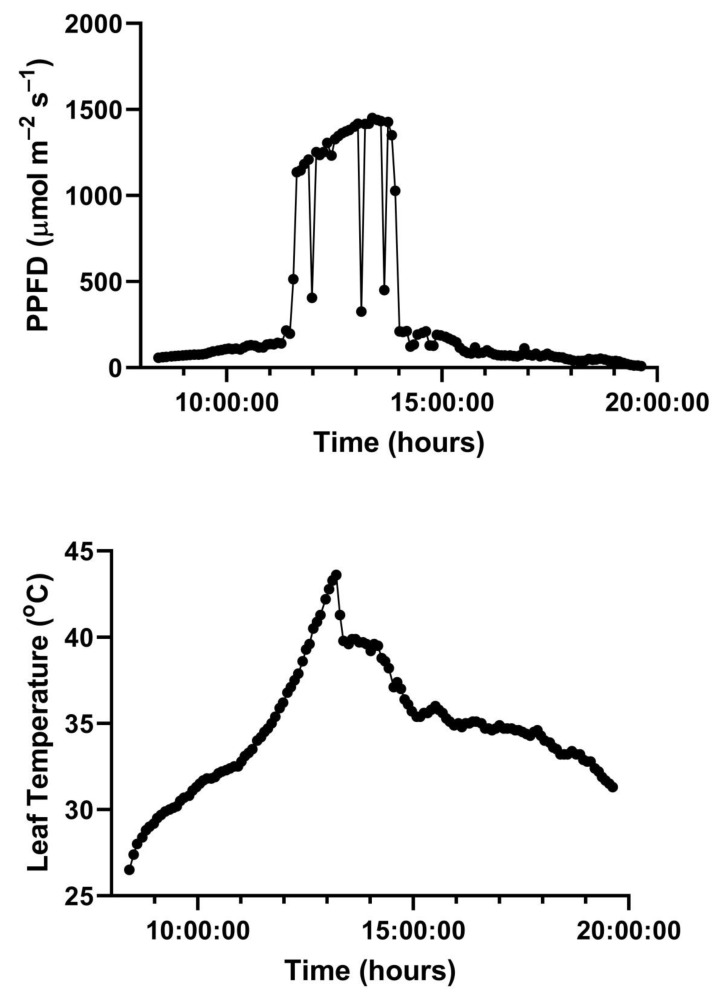

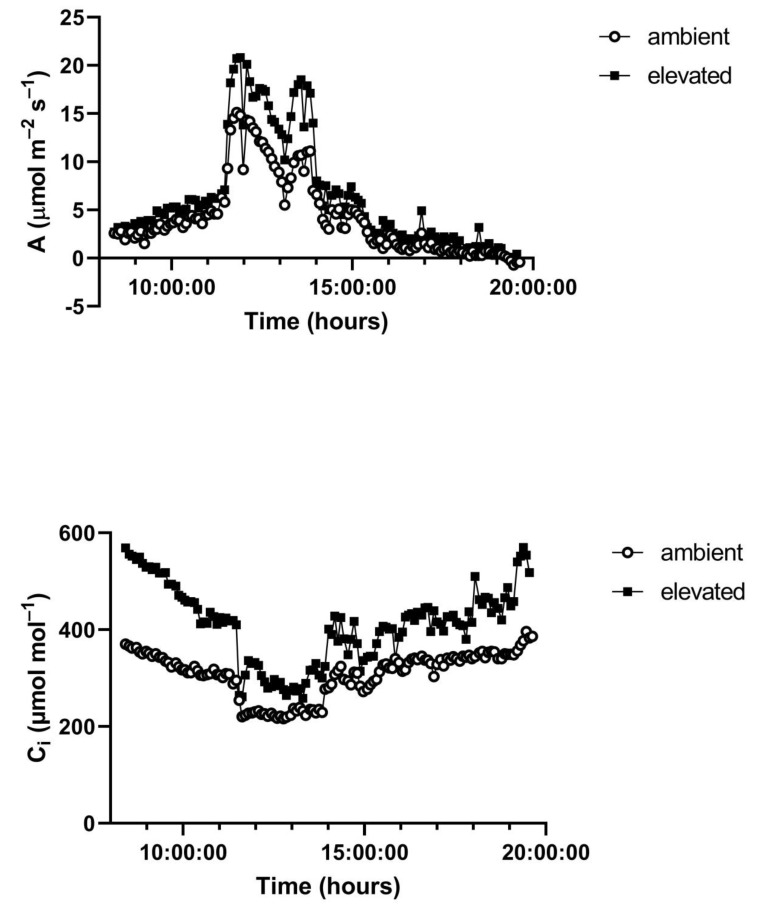
Diurnal patterns of PPFD, leaf temperature, A, and C_i_ of paired leaves of *H. fulva* kept at 400 (ambient) and 600 (elevated) μmol mol^−1^ CO_2_. Environmental variables are given for only one of the two leaflets, for clarity. See text for details.

A representative diurnal time course of environment and leaf gas exchange for leaves of *G. max* at 400 and 600 μmol mol^−1^ CO_2_ is presented in Figure 3. Representative diurnal time courses for the other two species are presented in Figure 4 and Figure 5. Mean daytime stomatal conductances to water vapor were 500 and 450 mmol m^−2^ s^−1^ for *G. max* at 400 and 600 mmol mol^−1^ CO_2_, respectively, and 120 and 100 mmol m^−2^ s^−1^ in *L. purpureus*, and 125 mmol m^−2^ s^−1^ at both CO_2_ levels in *H. fulva*. Values of stomatal conductance at any time point can be obtained from the values of A and Ci at that point. Because photosynthesis was modeled based on measured values of Ci, impacts of stomatal conductance on photosynthesis are incorporated in the analysis.

Because no differences occurred between morning and afternoon time periods in the outcome of the modeling of assimilation, either at the stable low or stable high PPFD periods, or after sudden decreases in PPFD due to clouds, values averaged over mornings and afternoons are presented in Table 1. The data reported in Table 1 for stable PPFD conditions represent means for two measurements (morning and afternoon) per day for three days in the cases of *G. max* and *H. fulva*, and four days in *L. purpurea*. The data for the sudden decrease in PPFD are means for two time points per day for each species, with three days in the case of *G. max* and *H. fulva*, and four days in *L. purpurea*. The results indicate that for all three species, when PPFD was stable, A at 600 μmol mol^−1^ CO_2_ was accurately modeled by assuming limitation of A at both 400 and 600 μmol mol^−1^ CO_2_ by Vc rather than J (Table 1). This was true for both high and low PPFD measurements, despite the rates of A being much lower at the low PPFD. In contrast, after sudden decreases in PPFD, A at 600 μmol mol^−1^ CO_2_ was more accurately modeled by assuming limitation of A at both 400 and 600 μmol mol^−1^ CO_2_ by J rather than by Vc (Table 1). 

**Figure 6:**
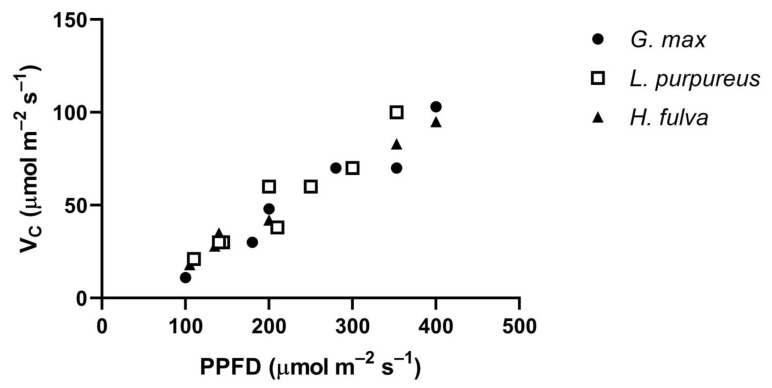
Vc measured at 30 to 35 °C at 400 μmol mol^−1^ CO_2_ at a range of low PPFD values, for three species. There were two morning and two afternoon measurements on three days in *G. max* and *H. fulva*, and four days in *L. purpureus*.

The Vc of all three species, measured at low PPFDs at 400 μmol mol^−1^ CO_2_, had similar approximately linear increases in Vc with PPFD (Figure 6). Leaf temperatures ranged from 30 to 35 °C in this comparison. These similar values among species at low PPFD levels occurred despite large differences among species in V_Cmax_, which ranged from about 160 μmol m^−2^ s^−1^ in *H. fulva* to 320 μmol m^−2^ s^−1^ in *G. max*, at 35 °C.

## 3. Discussion

The results of this study indicated that for gradual changes in PPFD over the course of a day, caused by changes in the solar angle, leaf photosynthesis was always limited by Vc rather than by J in all three of these C_3_ species, both at the approximate current ambient CO_2_ concentration and at 1.5 times the current concentration. Models which assume limitation of C_3_ photosynthesis by J at less than saturating PPFDs would underestimate the stimulation of daily CO_2_ fixation at 1.5 times the current CO_2_ concentration by approximately 50%.

The observed contrasting limitation of A by J after sudden decreases in PPFD caused by clouds obstructing direct solar radiation indicates that the assay of the type of limitation to photosynthesis used here was able to distinguish limitation by Vc from limitation by J. The change in type of limitation at low PPFD caused by the rate of decrease in PPFD is consistent with deactivation of Rubisco at low PPFD requiring a few to several minutes. Conducting rapid A *vs*. C_i_ curves [9,10] under field conditions may be a new alternative method of determining the biochemical limitations to photosynthesis throughout a day, although to date these seem to only have been conducted at saturating PPFD. However, field-based rapid A vs. C_i_ measurements would be much more labor intensive than the method used here, and would also require information on the operational C_i_ throughout the day.

Several field experiments have indicated that V_Cmax_ corrected to a constant temperature may change during the course of a day [11,12,13], although the cause of the changes is not clear, and can occur even in shade [13]. In those cases, V_Cmax_ was assayed using steady-state A vs. C_i_ curves at high PPFD, although it was not clear whether the assays allowed sufficient time at high PPFD to fully re-activate Rubisco. Clearly, models of C_3_ photosynthesis which assume constant V_Cmax_ throughout a day may be substantially in error. Furthermore, there is a notable lack of information on daily patterns of A vs. C_i_ curves at less than saturating PPFD, even at ambient CO_2_. This is particularly important in predicting responses of photosynthesis to past and future changes in atmospheric CO_2_, and may be one explanation for the observed insensitivity of biochemically based global photosynthesis models to past increases in atmospheric CO_2_ [6,14].

## 4. Materials and Methods

Three C_3_ species, *Glycine max* L. Merr., cultivar Clark, *Hemerocalus fulva* L., and *Lablab purpureus* L. Sweet were grown at the South Farm of the USDA Beltsville Agricultural Research Center, Beltsville, Maryland. The *G. max* and *L. purpureus* plants were grown from seed planted in the spring of 2019 in rows 70 cm apart, with plants thinned to about 7 cm between plants after emergence. The *H. fulva* plants were grown from tubers in rows 70 cm apart, with about 20 cm between tubers. The soil was a silt loam soil with a water table at about 1.5 m depth. Weeds were removed by hand. The prior crop was soybean, and no fertilizer was added to the soil after the well-fertilized soybean crop. Frequent precipitation prevented significant water deficits.

Two Ciras-3 portable photosynthesis systems (PP Systems, Amesbury, MA, USA) were used simultaneously each measurement day, with leaf chambers installed on opposite side leaflets of the same leaf in the case of *G. max* and *L. purpureus*, and both leaf chambers were on the same leaf in *H. fulva*, with about 5 cm between the chambers. Fully expanded, upper leaves were selected for measurement. Leaf chambers had circular windows 1.8 cm in diameter and were held horizontal. Leaf chambers were positioned so as to not be shaded by other leaves throughout the day. Leaf chambers were programmed to track ambient air temperature, and the external air temperature sensors were shaded at all times. Each chamber had internal PPFD sensors. The inlet air humidity setting was adjusted so that the chamber air had approximately the same water vapor content as that of the outside air. The CO_2_ concentrations of air streams entering the two chambers were controlled at 400 and 600 μmol mol^−1^. Leaf gas exchange data and environmental data were automatically recorded every 5 min from each leaf chamber throughout a 24 h period. Recording times of the two leaf chambers were not precisely synchronized, and the timing changed in each due to periodic automatic instrument self-tests, but recordings were synchronous within about 2 min. Measurements were begun soon after dew had evaporated from the leaves in the morning, and continued for 24 h. Measurements were continued for 24 h in order that rates in morning could be obtained without problems caused by dew on the leaves. Rates of respiration in darkness were not analyzed, for two reasons. First, flow rates were chosen based on obtaining accurate photosynthesis measurements, and created this very low, about 1 μmol mol^−1^, differentials in darkness, so respiration measurements would be quite imprecise. Secondly, measuring small CO_2_ differentials using clamp-on cuvettes has long been noted to produce artefactual apparent responses of respiration to CO_2_ concentration because of leakage [15]. 

Measurements were made on three days each for *G. max* and *H. fulva*, and four days for *L. purpureus*, with days randomly assigned to the species, and with random assignment of the two gas exchange systems to the two CO_2_ concentrations. All measurements were made in July and early August of 2019, on days selected as forecast to be mostly clear and without precipitation. All plants had flowered prior to the gas exchange measurements, but were plants were measured at least a month prior to reproductive maturity.

For each measurement day, detailed gas exchange analysis and modeling was applied to measurements during four periods: (1) periods of high (>1200 μmol m^−2^ s^−1^), stable PPFD before mid-day, (2) periods of high, stable PPFD after mid-day, (3) periods of low (100–300 μmol m^−2^ s^−1^), stable PPFD in early morning, and (4) periods of low, stable PPFD in late afternoon. Additionally, detailed analysis was made of periods of low (<500 μmol m^−2^ s^−1^) PPFD which occurred after PPFD had decreased by at least 700 μmol m^−2^ s^−1^ within the last 10 min, because of clouds. This later type of data was available each measurement day, which is typical for this climate, where intermittent afternoon clouds are very common in mid-summer.

Assimilation rates for the 400 μmol mol^−1^ treatment under each of the above conditions were used to estimate values of Vc and J which would be consistent with the measured values of A, C_i_, and temperature, using the FvCB model ([16], Equations (1) and (2)), with the temperature dependencies from Bernacchi et al. [17].

Anet = (1 − Γ*/Ci) × (Vc × Ci)/(Ci + Kc (1 + O/Ko)) − Rd(1)

Anet = (1 − Γ*/Ci) × (J × Ci)/(4Ci + 8Γ*) − Rd(2)

where Ci is intercellular (CO_2_), O is the oxygen concentration, γ* is the photosynthetic CO_2_ compensation point without dark respiration, Kc and Ko are the Michaelis–Menton constants for CO_2_ and O_2_, Vc is the carboxylation capacity of Rubisco, and J is the photosynthetic electron transport rate.

Mesophyll conductance was assumed to be infinite, so that the results would not be influenced by assumed finite values, given the lack of information about values of mesophyll conductance for *H. fulva* and *L. purpureus*, or its CO_2_ dependence [10]. It was then tested whether values of A at 600 μmol mol^−1^ taken at the approximately the same time points were better fit by assuming limitation of A by the values of Vc or J estimated from the leaves at 400 μmol mol^−1^. It could hypothetically occur that A at 400 μmol mol^−1^ would be limited by Vc while rates at 600 μmol mol^−1^ would be limited by J. That would lead to rates at elevated CO_2_ in between those predicted by limitation by Vc or by J [6], but that did not occur in these experiments.

## 5. Conclusions

Field leaf gas exchange measurements on three herbaceous species indicated that, under clear sky conditions, photosynthesis was limited by the carboxylation capacity of Rubisco rather than by electron transport rate throughout diurnal cycles of solar radiation both at approximately the current atmospheric CO_2_ concentration and at 1.5 times the current concentration. Abrupt decreases in radiation due to clouds temporarily resulted in limitation by electron transport capacity.

## Figures and Tables

**Table 1:** CO_2_ assimilation rates (A) of leaves measured at ambient (400 μmol mol^−1^) and elevated (600 μmol mol^−1^) CO_2_ at stable, high PPFD (>1200 μmol m^−2^ s^−1^), stable low PPFD (<400 μmol m^−2^ s^−1^), or within 10 min after PPFD abruptly decreased by at least 700 μmol m^−2^ s^−1^ due to clouds. Also presented are assimilation rates at elevated CO_2_ modeled using the model parameters fitted to the measured rates at ambient CO_2_, assuming limitation of rates at elevated CO_2_ either by V_C_ or by J at elevated CO_2_. See text for details.

		Measured A (μmol m^−2^ s^−1^)		Modelled A (Elevated)		
Species	PPFD	A Ambient	A Elevated	A V_C_	A J	Mean T (°C)
*G. max*	Stable high	29.3	43.5	43.3	36.1	37
	Stable low	8.7	14.1	14.2	12.0	33
	Sudden low	10.3	15.5	20.0	15.5	37
*H. fulva*	Stable high	11.2	20.4	20.6	16.1	37
	Stable low	5.1	6.6	6.7	6.0	33
	Sudden low	4.9	6.5	7.3	6.3	35
*L. purpurea*	Stable high	13.1	20.1	20.5	16.8	35
	Stable low	3.1	4.4	4.6	3.8	30
	Sudden low	2.5	3.0	4.0	3.2	37

## Data Availability

Data are available from the author upon request.
